# Progenitor Hematopoietic Cells Implantation Improves Functional Capacity of End Stage Coronary Artery Disease Patients with Advanced Heart Failure

**DOI:** 10.1155/2016/3942605

**Published:** 2016-04-11

**Authors:** Yoga Yuniadi, Yuyus Kusnadi, Lakshmi Sandhow, Rendra Erika, Dicky A. Hanafy, Caroline Sardjono, R. W. M. Kaligis, Manoefris Kasim, Ganesja M. Harimurti

**Affiliations:** ^1^Department of Cardiology and Vascular Medicine, Faculty of Medicine, University of Indonesia and National Cardiovascular Center Harapan Kita, Jakarta 11420, Indonesia; ^2^Stem Cell and Cancer Institute, Jakarta 13210, Indonesia

## Abstract

*Background*. Proangiogenic Hematopoietic Cells (PHC) which comprise diverse mixture of cell types are able to secrete proangiogenic factors and interesting candidate for cell therapy. The aim of this study was to seek for benefit in implantation of PHC on functional improvement in end stage coronary artery disease patients with advanced heart failure.* Methods*. Patients with symptomatic heart failure despite guideline directed medical therapy and LVEF less than 35% were included. Peripheral blood mononuclear cells were isolated, cultivated for 5 days, and then harvested. Flow cytometry and cell surface markers were used to characterize PHC. The PHC were delivered retrogradely via sinus coronarius. Echocardiography, myocardial perfusion, and clinical and functional data were analyzed up to 1-year observation.* Results*. Of 30 patients (56.4 ± 7.40 yo) preimplant NT proBNP level is 5124.5 ± 4682.50 pmol/L. Harvested cells characterized with CD133, CD34, CD45, and KDR showed 0.87 ± 0.41, 0.63 ± 0.66, 99.00 ± 2.60, and 3.22 ± 3.79%, respectively. LVEF was improved (22 ± 5.68 versus 26.8 ± 7.93, *p* < 0.001) during short and long term observation. Myocardial perfusion significantly improved 6 months after treatment. NYHA Class and six-minute walk test are improved during short term and long term follow-up.* Conclusion*. Expanded peripheral blood PHC implantation using retrograde delivery approach improved LV systolic function, myocardial perfusion, and functional capacity.

## 1. Introduction

Heart failure is one of the major health problems worldwide [1]. Myocardial cells damage during myocardial infarction results in the reduction of left ventricle systolic function which leads to heart failure [2]. Even in this era of advanced heart failure treatment, its survival rate remains low estimated at 50% and 10% in 5 and 10 years, respectively [3–5]. Furthermore, there was only about 12 percent decrease in death rate from heart failure per decade over the past fifty years [6, 7].

Cell therapy has been used as regenerative therapy in acute myocardial infarction and heart failure patient with promising results [8–10]. Myocardial regeneration potentially improves heart failure prognosis by increasing the number of functioning myocytes and enhances angiogenesis as well as the role of paracrine effects. Most clinical stem cell studies to date have been performed by using total bone marrow mononuclear cells which comprise hematopoietic progenitor cells, mesenchymal stem cells, and monocytes.

Endothelial progenitor cells (EPCs) have been a candidate that shows a therapeutic capacity to help treat heart failure. However, the identification of EPC remains controversial due to lack of firm consensus on its definition and classification. In most recent publication, EPCs are classified into Proangiogenic Hematopoietic Cells (PHC) and Endothelial Colony Forming Cells (ECFC) [11]. Proangiogenic Hematopoietic Cells which comprise diverse mixture of cell types including monocytes, macrophages, and hematopoietic progenitor cells are able to secrete proangiogenic factors. This study is aimed at seeking benefit of implantation of PHC derived from peripheral blood to induce functional improvement in end stage coronary artery disease patients with advanced heart failure and no other therapeutic option.

## 2. Methods

### 2.1. Patients

Thirty patients, aged from 18 to 75 years, with advanced heart failure participated in this study. All patients were still symptomatic (New York Heart Association [NYHA] Class of II or more) despite the guideline directed medical therapy (GDMT). To be included in this study patient must have left ventricle ejection fraction (LVEF) of 35% or less as measured by echocardiography. Patients with acute heart failure as well as cardiogenic shock, kidney, or liver failure were excluded from this study. Exclusion criteria also included past history of neoplasm or malignancies. Female patients must not be pregnant or lactating. Those who fulfilled the requirements would need to complete the patients consent form. The study was conducted in compliance with regulations by the ethics committee.

### 2.2. Preimplant Examination

Preimplant examination comprised assessment of left ventricle systolic function with Simpson's method of echocardiography examination, assessment of myocardial perfusion with Technetium (Tc) 99M Sestamibi scanning, assessment of heart failure biomarker, and assessment of functional status with NYHA classification and standard six-minute walk test.

### 2.3. Follow-Up

Data were collected based on short term (up to three months) and long term (up to one year) follow-up following PHC implantation. Repeat assessment of echocardiography and six-minute walk test are performed any time during short and long term follow-up. Perfusion scanning is performed only before and 6 months after stem cell implantation.

### 2.4. Collection of Peripheral Blood Sample

To avoid hemodynamic compromise, peripheral blood sample is collected in three divided times on three consecutive days. Only 80 mL of blood is collected at each time. An additional volume of 35 mL of peripheral blood was also taken on the first day to obtain the serum required as component of the growth medium of cells. Blood sample was then transported to the laboratory where isolation of mononuclear cells and cultivation of PHC would be performed.

### 2.5. Isolation, Cultivation, and Preparation of PHC

Mononuclear cells were isolated ex vivo out of 80 mL of peripheral blood each day by Ficoll density-gradient centrifugation as previously described [12, 13]. These cells were suspended in growth medium consisting of X-Vivo-15 (Lonza), 20% patient's serum, 1 ng/mL human recombinant vascular endothelial growth factor (VEGF) (R&D), and 0.1 *μ*mol/L atorvastatin (Pfizer). Cells were subsequently seeded at a density of 5.8 × 10^5^ cells/cm^2^ for the first day of blood collection and at a density of 8.7 × 10^5^ cells/cm^2^ for the second and third day of blood collection, in 2 self-coated fibronectin culture plates. The isolated mononuclear cells were also grown in 96-well plates under identical conditions to be used in the characterization of cells on the harvesting day.

On the fifth day after the first blood collection, adherent cells were harvested. Adherent cells were detached using 0.5 mmol/L ethylenediaminetetraacetic acid (EDTA). The harvested cells were then washed twice and resuspended in X-Vivo-10 to reach a final volume of 10 mL. This ready-to-use suspension contains heterogeneous population of progenitor cells which was then transported back to the hospital for implantation.

### 2.6. Characterization of PHC

#### 2.6.1. Immunophenotyping

Flow cytometry with combinations of antibody for cell surface markers including CD34, CD45, CD133, and kinase insert domain receptor (KDR) was used to characterize PHC. The following approach was used: 1 million cells were set aside from the total harvested cells and transported to the analytical laboratory. These cells were then washed with 10 mL PBS/2% FBS twice before being resuspended in the same solution and added with FcR blocker. Following 15 minutes of incubation at room temperature, cells were treated with different combinations of antibodies which would be the marker of the flow cytometry analysis performed afterwards. All reactions were carried out on ice.

#### 2.6.2. Functional Assays

As an alternative approach to determine the phenotype of PHC, functional assay was performed. Adherent cells from 96-well plate were added with Dil-acetylated-LDL, fixated with 3% paraformaldehyde, treated with UEA-1 lectin, and subjected to staining with DAPI consecutively with appropriate incubation and washing with PBS in-between staining. Fluorescence was observed with fluorescence microscope in the dark.

### 2.7. Retrograde Delivery Approach

**Figure 1:**
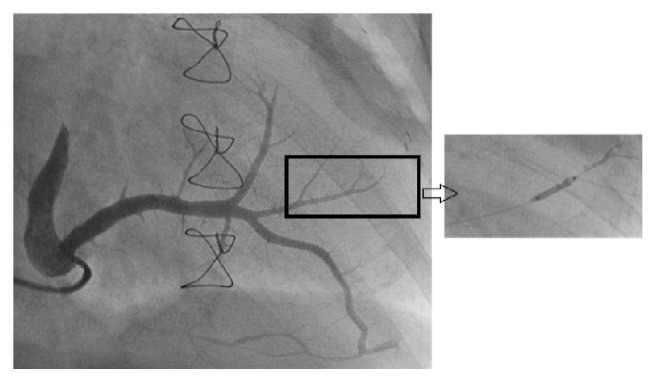
Retrograde delivery. Coronary sinus is cannulated using AL-1 catheter. Venogram shows anterolateral vein branches associated with infarcted area. The vein branch which is end vessel is selected as target vein. End vessel is confirmed by contrast injection through inflation over the wire balloon which shows no collateral (inset). Stem cells were delivered via the same balloon which is kept inflated for 15 minutes afterward.

The progenitor cell solution was delivered into the heart via sinus coronarius tributaries. Cannulation of coronary sinus was performed using Attain Command*™* sheath (Medtronic) via right subclavian or internal jugular vein. As the majority of patients suffered from anterior myocardial infarction, posterolateral or anterolateral vein was the target site selected for progenitor cell implantation. Target vein was wired using soft tip angioplasty wire. The vein branch without collateral to other veins (end vessel) was chosen. The particular vein branch was selected by injection of contrast media through OTW (over the wire) balloon to visualize whether collateral existed or not (Figure 1). After the completion of stem cell injection, the balloon remained inflated (6-7 atmosphere) up to fifteen minutes.

### 2.8. Perfusion Scanning

Technetium (Tc) 99m Sestamibi tracer is used to examine myocardial perfusion before and after eEPCs implantation. Using a score that represents perfusion for each of the multiple segments of the myocardium visual analysis is performed semiquantitatively. A segmentation model has been standardized for this approach by dividing the myocardium into 17 segments on the basis of three short axis slices and a representative long axis slice to depict the apex [14]. Perfusion was graded within each segment on a scale of 0 to 4, with 0 representing normal perfusion and 4 representing a very severe perfusion defect. Scores for all 17 segments were added to create a “summed” score. The sum of the segmental scores from the stress images (the Summed Stress Score (SSS)) represents the extent as well as the severity of stress perfusion abnormality and the magnitude of perfusion defects related to both ischemia and infarction. The sum of the 17 segmental scores from the rest images (the Summed Rest Score (SRS)) represents the extent of infarction. The summed difference score (SDS) was derived by subtracting the SRS from the SSS and represents the extent and severity of stress-induced ischemia [15].

### 2.9. Statistical Analysis

Clinical data collected for four weeks and three months after treatment were combined and analyzed as one variable which was called short term. Meanwhile, those obtained in six months and one year after injection were categorized as long term. Continuous variables were tested for normal distribution using Kolmogorov-Smirnov test and expressed as mean ± SD. Comparisons of normalized data between different times points were performed using paired Student's *t*-test while those without normal distribution were analyzed with nonparametric Wilcoxon test. Chi-squared test was used for categorical variables. Comparisons between different patient categories were done by using independent sample Student's *t*-test. SPSS Version 19.0 (SPSS Inc., Chicago, IL) was used to perform all statistical analysis. A *p* value of less than 0.05 was considered to be statistically significant.

## 3. Results

### 3.1. Clinical Characteristics

The data reported in this study consisted of results from 26 patients whose data were sufficiently complete to establish meaningful conclusions. All patients suffered from ambulatory heart failure as indicated by high NT proBNP level. Majority of subjects have severe three-vessel disease and suffered from diabetes mellitus (Table 1). Based on their clinical history, 9 patients were identified to suffer from coronary artery disease (CAD) while the remaining 15 were grouped under myocardial infarction (MCI) category. Baseline comparison between these two groups showed homogeneity in almost all variables except that for hsCRP which were significantly higher in MCI group. Sixteen patients who were diagnosed with diabetes mellitus showed no significant difference of clinical characteristics compared to those without diabetes mellitus, except for NT proBNP which was higher in diabetes group (1682.75 ± 789.255 versus 6501.2 ± 4908.402, *p* = 0.013).

### 3.2. Cellular Characteristics

Average total cell counts after 5-day expansion were more than 16 million cells with more than 92% of cells viability. CD45 was expressed by approximately 99.0 ± 2.60% of total population, making it the most dominantly expressed marker. Meanwhile, the other markers were low (0.87 ± 0.41%, 0.63 ± 0.66%, and 3.22 ± 3.79% for CD133, CD34, and KDR, resp.). The described cell marker proportion indicates that the cells possessed characteristic of hematopoietic cells. There was no difference in total cell number between CAD and MCI groups and between those with and without diabetes mellitus.

**Figure 2:**
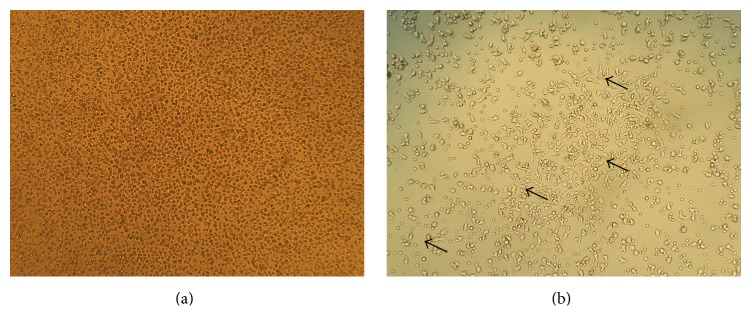
Observation of the cultured PMNCs. On day 1, the cells still display morphology resembling mononuclear cells (a). After 5-day culture, some of the cells exhibit spindle-like morphology (arrow), indicating that the cells are becoming more eEPCs like (b). Original magnification 100x.

**Figure 3:**
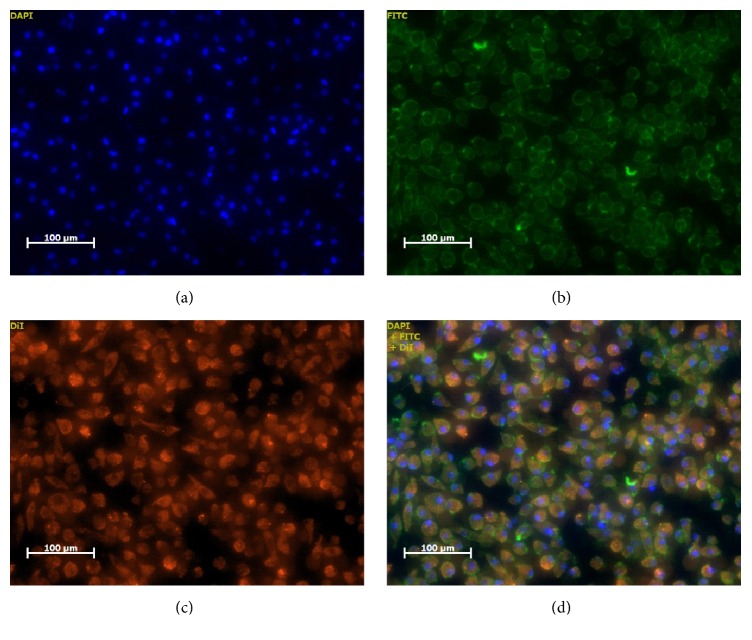
After 5 days of culture in the presence of VEGF and statin, the cells were stained with DAPI (a), UEA-1 lectin (b), Dil-acetylated LDL (c), and merge image (d). The cells exhibit the ability to bind lectin and uptake ac-LDL, one of the characteristics of eEPCs. Original magnification ×100.

On day 5 of culturing, three characteristics changes are observed. The first change is some PBMNCs morphology changed to more spindle-shaped cells which resembled more of eEPCS (Figure 2). The second change is functional assays result that revealed ability to uptake DiI-acetylated LDL and to bind to FITC-labeled lectin (UEA-1) favor of endothelial characteristic. The third change is cell's nuclei visible in blue-colored fluorescent of DAPI stain (Figure 3). Cells showing all the above three properties represent the phenotype found in endothelial cells, indicating the possibility that these cells have proangiogenic ability.

### 3.3. Follow-Up Data

Short and long term clinical data obtained during the scheduled follow-up sessions were compared with pretreatment data. It has been shown that there were significant improvements in echocardiography parameters that included both left and right ventricle systolic function as measured by LVEF and TAPSE (Table 2). The improvements of functional capacity are represented by increasing distance during the six-minute walk test and decreasing NYHA functional classification (Table 3). NT proBNP and hsCRP were reduced during short and long term observation but did not reach statistical significance (Table 4). However, the long term values were not significantly different from the short term values in all above parameters except for TAPSE.

**Figure 4:**
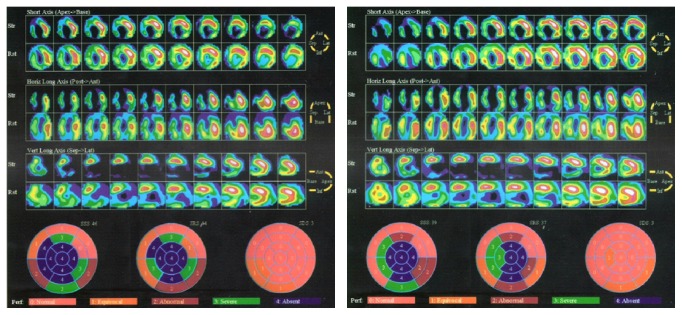
Myocardial perfusion. Left and right panel represent pre- and post-stem cell implantation status, respectively. Upper panels are visual interpretation that comprise short axis view, horizontal long axis view, and vertical long axis view during stress (Str) and rest (Rst). Lower panels are semiquantitative interpretation. Post-stem cell implantation shows more massive perfused myocard as presented with green and yellow color. Semiquantitative interpretation shows less area with score of 4 meaning less area with absent perfusion or nonviable cells. The Summed Stress Score (SSS) and Summed Rest Score (SRS) reduced after stem cell implantation.

Myocardial perfusion scanning study using Technetium (Tc) 99M Sestamibi was performed before and six months after stem cell implantation. All perfusion data of automated quantification either at resting state or under stress showed significant improvement (Table 5). Visual perfusion improvement was showed in Figure 4. Reduction of Summed Stress Score (SSS) and Perfusion Defect Size Stress (PDSS) after stem cell implantation indicated improvement of myocardial perfusion and reduction of perfusion defect. Reduction of Summed Rest Score (SRS) and Perfusion Defect Size Rest (PDSR) indicated reduction of nonviable tissue or increasing of viable tissue.

There were no significant differences of biomarker, echocardiography parameters, perfusion scan, and functional improvement between CAD and MCI groups. Comparing subjects with and without diabetes mellitus also shows no significant difference of the above-mentioned parameters, except for short term hsCRP marker which was lower in nondiabetic subjects.

There were total of 32 hospitalizations a year before study enrollment which reduced to only totally 6 hospitalizations during one-year follow-up. There was no death during one-year follow-up as well.

## 4. Discussion

This study showed that PHC implantation using retrograde delivery approach improved the LV systolic function, myocardial perfusion, and functional capacity. These promising results might give additional therapeutic alternative in symptomatic ischemic cardiomyopathy patients who have no other feasible therapeutic option.

### 4.1. PHC Characteristics

Our FACS results showed a low expression of EPC marker such as KDR, CD34, and CD133 but a high expression of CD45 indicating hematopoietic cells. However, our cells also exhibit positive results for ac-LDL uptake and lectin binding which implies the phenotype of endothelial cells. The low expression of EPCs markers on the positive ac-LDL uptake and lectin binding shows that these cells may be more resembling PHC instead of ECFC. Proangiogenic Hematopoietic Cells mainly comprise monocyte/macrophage derived cells but they possibly contained small proportion of true stem/progenitor cells and endothelial cells [11, 16, 17]. Proangiogenic Hematopoietic Cells promote angiogenesis via paracrine effects, in contrast to ECFC which have the capability to form new endothelium. Proangiogenic Hematopoietic Cells which comprise diverse mixture of cell types including monocytes, macrophages, and hematopoietic progenitor cells are similar to early EPCs characterized by Yoder from adherent cells in petri dish [18].

Surprisingly, autologous stem cells derived from peripheral blood of patients with advanced heart failure with severe coronary artery disease and multiple risk factors resulted in significant improvement of clinical parameters. The composition of medium used for ex vivo cultivation of cells and duration of culture influence the surface profiling of cells produced. In our protocol where statin was incorporated into the growth medium continuously, the number of endothelial progenitor colonies isolated from mononuclear cells was expected to improve [19]. The mechanisms of statin related stem cell functions improvement are increasing number of EPCs, accelerating reendothelialization, and reducing neointimal formation [19–21]. Ex vivo culturing of EPCs led to “uncapping” of telomeres, indicated by the loss of TRF2. Statin cotreatment of EPCs prevents impairment of their functional capacity by a TRF2-dependent, posttranscriptional mechanism [19].

### 4.2. Retrograde Approach

The delivery route of stem cell implantation might be another important key of beneficial effect in this study. Using retrograde approach through coronary vein system, stem can be delivered adjacent to infarcted area without facing any of the resistance from arteriole in scar area. In contrast to the transendocardial approach, in which cells are injected perpendicularly into the left ventricular wall, the transcoronary venous approach allowed parallel cell injection, which might result in greater cell retention [22]. This delivery method did not produce hemodynamic changes and reported enhanced angiogenesis and observed autologous bone marrow stem cells in the myocardium [23–25].

### 4.3. On Short versus Long Term Data

Significant improvement of functional capacity and LV systolic function was achieved during short term follow-up and persisted during long term follow-up. However, there was no significant difference between short and long term improvement. Using autologous bone marrow mononuclear cells previous studies showed similar results of improved quality of life and exercise capacity during short and long term follow-up [26, 27]. The pattern of clinical improvements consists of peak initial short term improvement which is maintained during long term observation [27]. The persistent beneficial hemodynamic effects in the long term justified the assumption that PHC implantation might overcome the possible detrimental effects of ventricular remodelling [27].

During one-year follow-up, hospitalization rate was significantly reduced and no death was observed in our patients. Similar to our results, recent meta-analysis of thirteen trials reported no death during their follow-up and five trials reported significant lower rehospitalization rate due to worsening of heart failure among patients receiving cell therapy [28].

### 4.4. Myocardial Perfusion

Significant improvement of myocardial perfusion had been shown six months after stem cell implantation. Technetium (Tc) 99M Sestamibi was used to examine myocardial perfusion and viability. Technetium (Tc) 99M Sestamibi is a cationic Tc 99M complex which has been found to accumulate in viable myocardial tissue with less soft-tissue attenuation, better counting statistics, and improved image quality with single-photon emission CT (SPECT) [29]. This study utilized automated quantification of myocardial perfusion and showed that SSS, SRS, PDSS, and PDSR value were improved after stem cell implantation. Reduction of SSS and PDSS after stem cell implantation indicates improvement of myocardial perfusion and reduction of perfusion defect. Reduction of SRS and PDSR indicates reduction of nonviable tissue or increasing of viable tissue. These findings employ therapeutic nature of ex vivo cultivated PHC in the treatment of cardiovascular diseases. The perfusion improvement may not be achieved by incorporation of PHC to form new endothelium but is more likely due to proangiogenic factors secreted by implanted PHC such as vascular endothelial growth factor (VEGF), hepatocyte growth factor (HGF), granulocyte colony stimulating factor (GCSF), and granulocyte-macrophage colony stimulating factor (GMCSF) [16]. This secretome then helps the recruitment of native EPCs and maintains their proliferation and survival. Moreover, although it is not proven in this study, there might be a very small population of ECFC in our culture that may contribute to vasculogenesis [16, 17].

### 4.5. Limitation

Our study used a simple FACS panel to characterize PHC and the growth factors secreted by our cells were not analyzed. Further studies should apply a more comprehensive FACS panel to make sure what kind of cells are cultured and a range of growth factors ought to be assessed to measure the proangiogenic capacity of PHC.

In this pre-post study, control group and experimental group were of same patients. Even though all patients were in stable condition with fixed GDMT before study enrollment, some changes might happen during the course of study.

## 5. Conclusion

Implantation of peripheral blood PHC resulted in clinical and functional improvement in end stage coronary artery disease with advanced heart failure patients. The study shows potential clinical efficacy and gives a basis for future studies with a larger number of patients.

## Figures and Tables

**Table 1:** Clinical characteristics.

Variable	Results
Age (years)	56.4 ± 7.40
Sex M/F (*n*)	25/1
Angiography [*n* (%)]	
One VD	4 (15)
Two VD	3 (12)
Three VD ± LMD	19 (73)
Myocardial infarction [*n* (%)]	17 (65)
Diabetes mellitus [*n* (%)]	15 (58)
Creatinine	1.2 ± 0.29
Hemoglobin	12.7 ± 3.13
Hematocrit	37.8 ± 9.23
Leukocyte	6748 ± 1959.48
hsCRP	7.5 ± 8.39
NT proBNP	5124.5 ± 4682.50

VD: vessel disease, hsCRP: high sensitive C reactive protein, and NT proBNP: N-terminal prohormone of brain natriuretic peptide, LMD: left main disease.

**Table 2:** Echocardiography parameters.

Parameters	Baseline	Short term	Long term	*p* value (baseline to short term)	*p* value (baseline to long term)	*p* value (short to long term)
LV EF (%)	22 ± 5.68	26.8 ± 7.93	26.9 ± 10.72	<0.001	0.014	0.983
LV EDD (mm)	66.0 ± 8.60	64.8 ± 8.10	66.0 ± 9.28	0.331	0.975	0.302
LV ESD (mm)	58.7 ± 9.74	56.1 ± 9.79	57.4 ± 18.76	0.079	0.408	0.373
TAPSE	1.3 ± 0.41	1.5 ± 0.42	1.6 ± 0.44	0.018	<0.001	0.019

LV: left ventricle, EF: ejection fraction, EDD: end diastolic dimension, ESD: end systolic dimension, EDV: end diastolic volume, ESV: end systolic volume, and TAPSE: tricuspid annular presystolic excursion.

**Table 3:** Functional capacity parameters.

Parameters	Baseline	Short term	Long term	*p* value (baseline to short term)	*p* value (baseline to long term)	*p* value (short to long term)
NYHA Class	2.16 ± 0.69	1.2 ± 0.38	1.2 ± 0.34	<0.001	<0.001	0.327
6 WT						
(i) Distance	297.8 ± 91.63	345 ± 73.94	351.9 ± 89.77	0.006	0.006	0.519
(ii) METS	5.2 ± 1.74	6.0 ± 1.46	6.2 ± 1.79	0.012	0.004	0.130

NYHA Class: New York Heart Association Classification and 6 WT: six-minute walk test.

**Table 4:** Laboratory parameters.

Parameters	Baseline	Short term	Long term	*p* value (baseline to short term)	*p* value (baseline to long term)	*p* value (short to long term)
NT proBNP	5124.5 ± 4682.50	3235.1 ± 2190.74	3092 ± 2079.75	0.101	0.087	0.858
hsCRP	7.5 ± 8.39	7.1 ± 9.33	5.3 ± 8.53	0.877	0.084	0.142

hsCRP: high sensitive C reactive protein and NT proBNP: N-terminal prohormone of brain natriuretic peptide.

**Table 5:** Perfusion data.

	SSS	SRS	PDSS	PDSR
Before	26.6 ± 12.45	26.2 ± 11.507	53.88 ± 19.32	50.16 ± 18.929
6 months	18.32 ± 16.09	17.8 ± 15.521	34.8 ± 29.165	33.28 ± 28.6
*p* value	0.007	0.006	0.003	0.004

PDSR: Perfusion Defect Score Rest; PDSS: Perfusion Defect Score Stress; SRS: Summed Rest Score; SSS: Summed Stress Score.

## Abbreviations

CHF: Chronic heart failure
LVEF: Left ventricle ejection fraction
CAD: Chronic artery disease
MI: Myocardial infarction
EDTA: Ethylenediaminetetraacetic acid
FBS: Fetal bovine serum
GDMT: Guideline directed medical therapy
LDL: Low density lipoprotein
DiI: 1,1′-Dioctadecyl-3,3,3′3′-tetramethylindocarbocyanine perchlorate
UEA-1: Ulex Europaeus Agglutinin-1
eEPCs: Early Endothelial Progenitor Cells
VD: Vessel disease
EF: Ejection fraction
METS: Metabolic equivalents
TAPSE: Tricuspid annular plane systolic excursion
hsCRP: High sensitivity C reactive protein
SCD: Sudden cardiac death
KDR: Kinase insert domain receptor
DAPI: 4′6-Diamidino-2-phenylindole
PBS: Phosphate buffered saline
PDSR: Perfusion Defect Score Rest
PDSS: Perfusion Defect Score Stress
PMNCs: Peripheral mononuclear cells
VEGFR: Vascular endothelial growth factor receptor
SRS: Summed Rest Score
SSS: Summed Stress Score.
